# The International Prognostic Index for Patients with Chronic Lymphocytic Leukemia Has the Higher Value in Predicting Overall Outcome Compared with the Barcelona-Brno Biomarkers Only Prognostic Model and the MD Anderson Cancer Center Prognostic Index

**DOI:** 10.1155/2018/9506979

**Published:** 2018-03-15

**Authors:** Carolina Muñoz-Novas, María Poza-Santaella, Isabel González-Gascón y Marín, María Hernández-Sánchez, Ana-Eugenia Rodríguez-Vicente, María-Stefania Infante, Cecilia Heras, María-Ángeles Foncillas, Karen Marín, Jesús-María Hernández-Rivas, José-Ángel Hernández

**Affiliations:** ^1^Servicio de Hematología, Hospital Universitario Infanta Leonor, Madrid, Spain; ^2^Departamento de Medicina, Universidad Complutense de Madrid, Madrid, Spain; ^3^IBSAL, IBMCC, Centro de Investigación del Cáncer, Universidad de Salamanca-CSIC, Servicio de Hematología, Hospital Universitario de Salamanca, Salamanca, Spain

## Abstract

In recent years, new prognostic indexes (PIs) for chronic lymphocytic leukemia (CLL), which include clinical, biological, and genetic variables, have been validated, highlighting the MD Anderson Cancer Center prognostic index (MDACC PI), the CLL-international prognostic index (CLL-IPI), and the Barcelona-Brno biomarkers only prognostic model. The aim of this study is to compare the utility of these PIs in a cohort of Spanish patients. A retrospective analysis of 696 unselected CLL patients newly diagnosed and previously untreated from different Spanish institutions was performed. The MDACC PI, the CLL-IPI, and the biomarkers only PI were applied to these patients, and a comparison of the three PIs was performed. With a median follow-up time of 46 months, 394 patients were alive and 187 had received treatment. The median overall survival (OS) was 173 months and the median time to first therapy (TTFT) was 32 months. Significant differences were obtained in OS and TTFT for all subgroups when applying these PIs, with the CLL-IPI being the one with the higher *c*-index (0.676 for OS and 0.757 for TTFT). The three PIs were able to discriminate patients in different prognostic subgroups. In our cohort, the CLL-IPI showed higher power in predicting TTFT and OS.

## 1. Introduction

Chronic lymphocytic leukemia (CLL) is the most frequent leukemia in Western countries and is characterized by a marked clinical, molecular, and prognostic heterogeneity [1]. While some patients without treatment have a life expectancy equal to that of the healthy population, others require treatment from the beginning of the disease and may even die within a short period of time [2–4]. For this reason, more than 35 years ago, the Rai [5] and Binet [6] classifications appeared. Although these classifications are widely used, they have shown certain limitations in the ability to predict which patients will have a more aggressive progression and which ones will respond worse to treatment [1].

In recent years, many studies have been carried out to identify characteristics and biomarkers related to the tumor process and to the patient. From these results, it has been possible to establish new PIs that solve most of the limitations of classical staging systems. The advances in the identification of cytogenetic alterations analyzed by fluorescence in situ hybridization (FISH) are worth mentioning, which distinguish groups with favorable prognosis (13q deletion [13q-]) and unfavorable prognosis (11q deletion [11q-] or 17p deletion [17p-]) [7]. Likewise, the study of somatic mutations in the variable region of the immunoglobulin heavy chain gene* (IGHV)* has shown that patients with mutated pattern have favorable outcome [8–10].

In 2007, the MD Anderson Cancer Center (MDACC) [11] designed a nomogram out of a retrospective study of 1.674 patients diagnosed with CLL. Prognostic factors included were sex, age, absolute lymphocyte count, *β*2-microglobulin, Rai stage, and the number of nodal regions affected. Although this prognostic index (PI) has been validated extensively by other groups [12–18] and is very useful because of its simple application, its main limitation is that the parameters included are closely related to the tumor burden and not to genetic factors [19].

Recently, the chronic lymphocytic leukemia international prognostic index (CLL-IPI) [20], which combines genetic, biochemical, and clinical parameters in a prognostic model, has been published, based on the results of a meta-analysis and subsequently validated in other publications [13, 21, 22]. The CLL-IPI includes five variables (mutation or chromosomal status of the* TP53*/17p- gene, serum *β*2-microglobulin, mutation status of* IGHV*, Rai/Binet clinical stage, and age) and assigns a score according to their greater or lower prognostic impact.

With the aim to facilitate the use of the CLL-IPI in routine clinical practice, a simplified version of this PI, performed by Hospital Clinic Barcelona, University of Brno Hospital, Azienda Ospedaliera Pugliese-Ciaccio, and Azienda Ospedaliera di Cosenza groups, which only includes* IGHV *mutation status and FISH cytogenetics, has been proposed recently. It has shown a similar discriminatory value to the CLL-IPI and has been applied independently of age, separating patients with different risks among the same clinical stage groups [23].

The objective of this work is the evaluation of the validity and reproducibility of the CLL-IPI, the Barcelona-Brno biomarkers only prognostic model, and its comparison with the MDACC in a cohort of Spanish patients.

## 2. Materials and Methods

### 2.1. Patients

A total of 696 unselected CLL patients newly diagnosed and previously untreated from different institutions of the central region of Spain were included in this study. The data collection period began in 2004 and ended in 2014. This study was approved by the local ethics committee, and the ethical norms of the Declaration of Helsinki were followed.

The database contains information about demographic (age and sex), clinical (nodal regions affected, hepatomegaly, and splenomegaly), analytical (blood counts, LDH, and *β*2-microglobulin), and genetic abnormalities determined by FISH (11q-, trisomy 12, 13q-, and 17p-), immunophenotypic (CD38 and ZAP70 expression) and molecular (somatic mutations of the* IGHV* gene) variables, and Rai and Binet clinical stages. A review of the patients' medical records was carried out, and 107 cases were excluded of the analysis due to incomplete data. Finally, 483 patients were included to assess the MDACC PI and 258 for the CLL-IPI and the Barcelona-Brno biomarkers only prognostic model, as the remaining cases did not have information about the mutation status of the* IGHV* gene.

### 2.2. MDACC Prognostic Index

The classification in the three groups of risk proposed by the MDACC, low risk (1–3 points), intermediate risk (4–7 points), and high risk (≥8 points), was determined from the sum of the points assigned to six prognostic factors [11]. The index was calculated after assigning 1 point for age < 50 years, male sex, level of *β*2-microglobulin 1-2x upper limit of normality, absolute lymphocyte count of 20–50 × 10^9^/L, Rai stage III or IV, and ≥3 nodal regions affected; 2 points for age 50–65 years, *β*2-microglobulin >2x upper limits of normality, and absolute lymphocyte count >50 × 10^9^/L; and 3 points for age > 65 years.

### 2.3. CLL-IPI

In order to stratify patients according to the CLL-IPI, 4 points were assigned for 17p- mutation, 2 points for unmutated* IGHV* status and serum *β*2-microglobulin >3.5 mg/L, and 1 point for age > 65 years and advanced clinical stage (Rai I–IV or Binet B-C). As* TP53* mutational status was not available in the database, only 17p- was used to assess* TP53* status. The sum of these scores identified patients in 4 subgroups: low risk (0-1 points), intermediate risk (2-3), high risk (4–6), and very high risk (7–10) [20].

### 2.4. Barcelona-Brno Biomarkers Only (*IGHV* Mutational Status and FISH Cytogenetics Prognostic Model)

The simplified version of the CLL-IPI defined high-risk patients as those with adverse FISH cytogenetics (11q- and/or 17p-) and an unmutated* IGHV* status, low-risk patients as those without adverse cytogenetics and mutated* IGHV *status, and intermediate-risk patients as those not included in the previous groups [23].

### 2.5. Statistical Analysis

Statistical analysis was performed using the SPSS software package version 21.0. Overall survival (OS) was calculated from the time of diagnosis to death or last follow-up and time to first therapy (TTFT) from the date of diagnosis to first treatment or last follow-up. Both variables were estimated by the Kaplan-Meier method and assessed by the log-rank test. Cox regression was used for univariate and multivariate analyses of the impact of variables on OS. These data were expressed as the hazard ratio (HR) with a 95% confidence interval (95% CI). On the other hand, the area under the ROC (receiver's operating characteristic) curve was used to find the discrimination of models. In the same way, a 95% CI was established, in which 0.5 implies that the model offers random results and 1 implies that the model is a perfect predictor of survival. The value of *p* < 0.05 was considered significant for all analyses.

## 3. Results

### 3.1. Patients Characteristics

A total of 483 patients with CLL were included in the analysis of the MDACC prognostic index. The principal characteristics of these patients are shown in Table 1. Of note, most of the patients presented with early clinical Rai or Binet stages. The median age at diagnosis was 67 years (range: 25–90) and most patients were older than 50 (92.3%). After a median follow-up period of 46 months (range: 1–277), 92 individuals had died and 186 had required treatment. The median time to treatment was 32 months (range: 0–264).

The analysis for the validation of CLL-IPI and the Barcelona-Brno biomarkers only prognostic model included 258 patients.  Table 2 illustrates the main characteristics of this subgroup of patients. Analogously to the previous cohort, the majority of the patients presented with early Rai or Binet stages. In this cohort, the median follow-up period was 68 months (range: 3–277), during which 47 patients died and 113 were treated. The median period to the treatment was 31 months (range: 0–264).

### 3.2. Application of the MDACC Prognostic Index

**Figure 1:**
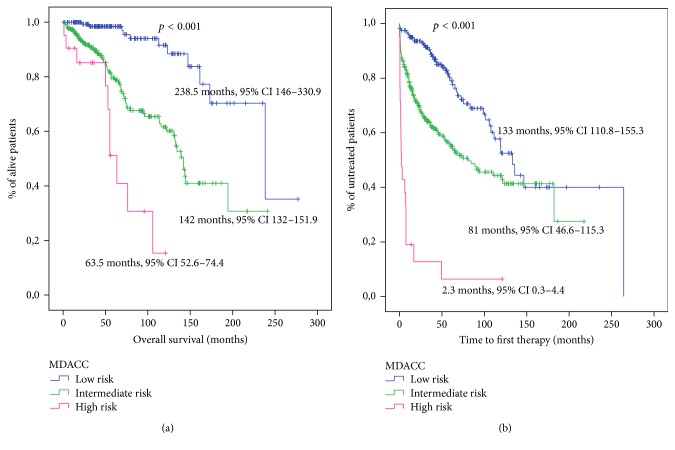
Survival analysis for patients stratified according to the MDACC score. (a) Overall survival analysis. (b) Time to first therapy analysis.

The distribution of patients in the three prognostic groups proposed by the index was 160 (33.1%) patients at low risk, 302 (62.5%) at intermediate risk, and 21 (4.4%) at high risk. This division of the patients proved to have a statistically significant association with OS (Figure 1(a)). The probability of 5-year survival and 10-year survival showed significant differences between risk groups (Table 3).

This index also showed a significant association with TTFT (Figure 1(b)). The probability of treatment at 5 years and 10 years is shown in Table 3.

### 3.3. Application of the CLL-IPI Prognostic Index

**Figure 2:**
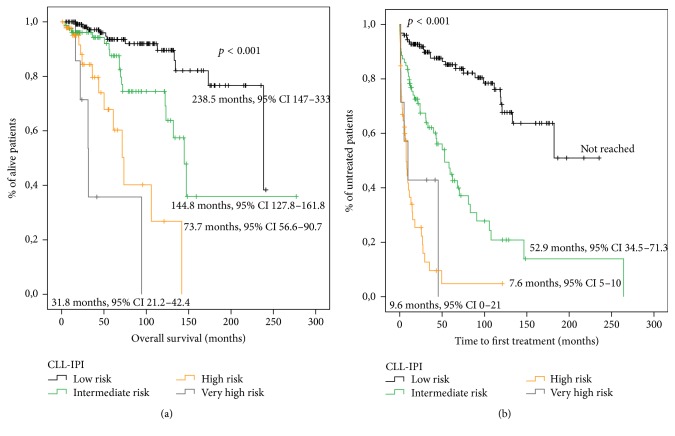
Survival analysis for patients stratified according to the CLL-IPI score. (a) Overall survival analysis. (b) Time to first therapy analysis.

Patients were divided into the four prognostic groups proposed by the CLL-IPI as follows: 126 (48.8%) at low risk, 79 (30.6%) at intermediate risk, 46 (17.8%) at high risk, and 7 (2.7%) at very high risk. The model proved to be statistically significant in the prediction of OS (Figure 2(a)). The probability of 5-year survival and 10-year survival showed significant differences between risk groups (Table 3).

The CLL-IPI also showed a statistically significant association with TTFT (Figure 2(b)). The probability of treatment at 5 years and 10 years is shown in Table 3.

Detailed univariate and multivariate Cox regression analyses of the variables included in the CLL PI are shown in Tables S1 and S2.

### 3.4. Application of the Barcelona-Brno Biomarkers Only Prognostic Model

**Figure 3:**
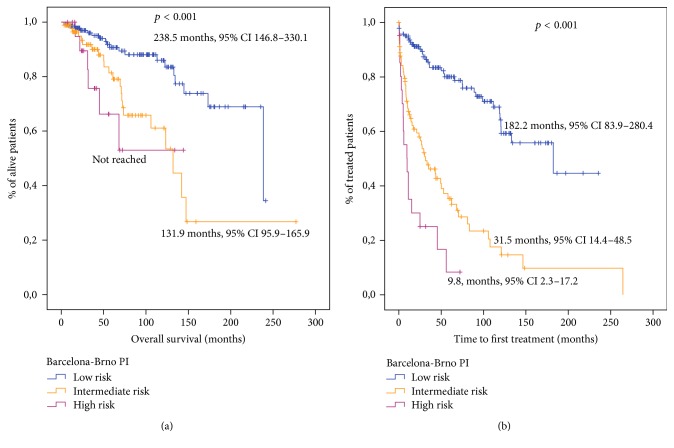
Survival analysis for patients stratified according to the Barcelona-Brno biomarkers only prognostic model. (a) Overall survival analysis. (b) Time to first therapy analysis.

The Barcelona-Brno biomarkers only prognostic model also distinguished 3 groups of patients with different OS: 145 (56.2%) patients at low risk, 91 (35.3%) at intermediate risk, and 22 (8.9%) at high risk (Figure 3(a)). The probability of 5-year survival and 10-year survival also showed significant differences between risk groups (Table 3). In addition, this index showed a statistically significant association with TTFT (Figure 3(b)). The probability of treatment at 5 years and 10 years is described in Table 3.

### 3.5. Comparison of the Three Indexes

The ROC curve to verify the discriminatory capacity of the index proposed by the MDACC showed a *c*-statistic value of 0.652 for OS and 0.614 for TTFT (*p* < 0.0001 in both). The *c*-statistic ROC curve of CLL-IPI was 0.676 for OS and 0.757 for TTFT (*p* < 0.0001 in both). The *c*-statistic of the Barcelona-Brno biomarkers only prognostic model for OS was 0.600 (*p* = 0.03) and for TTFT was 0.722 (*p* < 0.001).

The ROC curves are shown in Figures S1, S2, and S3.

## 4. Discussion

CLL is a disease with an extremely variable clinical outcome. Nowadays, despite the discovery of new drugs focused on new targets, CLL is not considered curable. Several PIs have been developed in previous years to refine the prognosis of CLL patients. The objective of these new scores is to apply them in daily clinical practice, to improve risk stratification and, as possible, to predict response to therapy. Currently,* TP53 *mutations/17p deletions and the mutational status of* IGHV* gene constitute the main predictive factors in CLL patients [23–25].

Therefore, in this study, performed with a representative sample of patients with CLL, the application of different previously published prognostic systems is analyzed. The characteristics of the patients included in the study are similar to previous publications [12, 13, 20, 26–28] and representative of the patients diagnosed in usual clinical practice. This made it possible to overcome the main limitation of the MDACC study by Wierda et al. [11], in which the median age at diagnosis was not clearly representative of patients with CLL (58 years old).

Our study has confirmed the ability of the three PIs to stratify patients according to its clinical outcome. The 5-year OS and 10-year OS obtained in the three cohorts of the study were similar to those published in the MDACC PI, CLL-IPI, and Barcelona-Brno biomarkers only prognostic model [11, 17, 18, 20, 23] except in the very high risk CLL-IPI group [20]. This fact may be due to the low number of patients classified in this subgroup and could also be explained by the absence of data about the mutation* TP53 *in this study. This mutation occurs around 10–15% in the absence of 17p deletion [29], so a small percentage of patients may not have been classified in the very high risk group in our study.

In the comparison of the three PIs in the prediction of OS, the AUC of the CLL-IPI was the higher (0.676 CLL-IPI versus 0.652 MDACC PI versus 0.600 Barcelona-Brno prognostic model). The same conclusion was reached in the prediction of TTFT (0.757 CLL-IPI versus 0.614 MDACC PI versus 0.722 Barcelona-Brno prognostic model). All this indicates that the precision in the prediction of OS and TTFT is higher in the CLL-IPI, suggesting that the incorporation of molecular and cytogenetic prognostic factors is relevant in the prognostic impact of these scores. Moreover, the Barcelona-Brno biomarkers only prognostic model was able to separate three different groups with different outcome, and, perhaps, it could be easier to use for the clinical practice in the near future. In addition, it has the advantage of the inclusion of patients with 11q- (associated with poor prognosis) [23], although in our series the most powerful PI was the CLL-IPI. However, our research confirms the results obtained in recent studies comparing these indexes and demonstrates the superiority of the CLL-IPI compared to the other models [13, 21, 30].

We also compared the ability of these indexes to predict TTFT in newly diagnosed patients. In this study, the three indexes have shown a statistically significant association with TTFT and were able to segregate patients with different TTFT (Figures 1(b), 2(b), and 3(b)).

A limitation of this study is that only 258 cases could be analyzed for the validation of the CLL-IPI, because the mutational status of* IGHV *was not yet available in all cases. However, a similar frequency in terms of age, sex, lymphocyte count, *β*2-microglobulin levels, Rai stage, and number of nodal regions affected appears to make this subgroup representative of the original sample.

Our study included patients diagnosed between 1989 and 2013, a period of great advances in the treatment of the disease. In this context, these PIs were validated with patients treated with chemotherapy or chemoimmunotherapy, so results cannot be generalized to patients treated with the new inhibitors of B cell receptor and Bcl-2 antagonists. These novel therapies have transformed the treatment for patients with CLL, especially in patients with higher risk, whom treatment individualization is essential [13, 31].

Recently, genetic mutations affecting* NOTCH1*,* SF3B1*,* MYD88*, and* BIRC3* genes have been discovered [27]. Alterations of these genes occur in approximately 5–10% of CLL patients at diagnosis and, in the case of* NOTCH1*,* SF3B1*, and,* BIRC3*, have shown significant correlations with poor survival in consecutive series. In this setting, dynamic model based on both chromosomal abnormalities detected by FISH and gene mutations has been proposed for Rossi et al. [32]. However, this PI is more complicated to perform, as next-generation sequencing technique implies significant cost and is not recommended in current CLL guidelines [33, 34]. Further studies are required to determine whether their applicability in current and future clinical practice is feasible.

In conclusion, the three PIs have a great ability to predict the clinical course of patients diagnosed with CLL. In addition, the incorporation of cytogenetic and molecular variables such as 17p-/*TP53 *mutation and the mutational state of* IGHV *adds an evident predictive gain. However, the index proposed by the MDACC should not be relegated, since, besides being easily applicable, it has been validated on several occasions [12–18].

## Figures and Tables

**Table 1:** Clinical characteristics of the patients included for the validation of the MDACC prognostic index.

	Patients (%)	Median (Q1, Q3)
*Age (years) *		67.4 (58.9–74.6)
<50	37 (7.7)	
50–65	172 (35.6)	
>65	274 (56.7)	
*Sex*		
Male	310 (64.2)	
Female	173 (35.8)	
*Lymphocyte count (×10* ^*9*^ */L)*		13.0 (8.6–22.6)
<20	346 (71.6)	
20–50	93 (19.3)	
>50	44 (9.1)	
*Hemoglobin (g/dL)*		14.3 (13.1–15.3)
≤10	22 (4.6)	
>10	461 (95.4)	
*Platelet count (×10* ^*9*^ */L)*		185 (145–226)
≤100	27 (5.6)	
>100	456 (94.4)	
*β2-microglobulin (mg/L) *		2.15 (1.6–3.0)
Normal	239 (49.2)	
1-2x ULN	184 (37.9)	
>2x ULN	63 (12.9)	
*LDH (UI/L) *(*n* = 401)		323 (271–393)
*Enlarged node regions *		
≤2	395 (81.9)	
>2	88 (18.2)	
*Splenomegaly*		
Yes	80 (16.6)	
Not	403 (83.4)	
*Hepatomegaly *		
Yes	35 (7.2)	
Not	448 (92.8)	
*Rai staging system*		
0-2	453 (93.8)	
3-4	30 (6.2)	
*FISH *		
Del11q	34 (7.0)	
Trisomy 12	57 (11.8)	
Del13q	192 (39.8)	
Del17p	21 (4.3)	
Normal	179 (37.1)	
*ZAP70 *(*N* = 183)		
Positive	29 (15.8)	
Negative	154 (84.2)	
*CD38 *(*N* = 338)		
Positive	79 (23.4)	
Negative	259 (76.6)	

Q1: quartile 1; Q3: quartile 3; ULN: upper limit normal; MDACC: MD Anderson Cancer Center.

**Table 2:** Clinical characteristics of the patients included for the validation of the CLL-IPI prognostic index.

	Patients (%)	Median (Q1, Q3)
*Age (years) *		65.7 (55.2–73.5)
<65	123 (47.9)	
≥65	274 (52.1)	
*Sex*		
Male	162 (62.8)	
Female	96 (37.2)	
*Lymphocyte count (×10* ^*9*^ */L) *		13.2 (9.13–23.6)
≤20	184 (71.3)	
>20	74 (28.7)	
*Hemoglobin (g/dL)*		14.3 (13.2–15.3)
≤10	9 (3.5)	
>10	249 (96.5)	
*Platelet count (×10* ^*9*^ */L)*		181 (145–226)
≤100	12 (4.7)	
>100	246 (95.3)	
*β2-microglobulin (mg/L) *		2.10 (1.6–2.8)
≤3.5	223 (86.4)	
>3.5	35 (13.6)	
*LDH (UI/L) *(*n* = 226)		340 (298–398)
*Enlarged node regions*		
≤2	205 (79.5)	
>2	53 (20.5)	
*Splenomegaly*		
Yes	52 (20.2)	
Not	206 (79.8)	
*Hepatomegaly *		
Yes	22 (8.5)	
Not	236 (91.5)	
*Rai staging system*		
0	152 (58.9)	
1–4	106 (41.1)	
*FISH *		
Del11q	20 (7.8)	
Trisomy 12	33 (12.8)	
Del13q	107 (41.5)	
Del17p	10 (3.9)	
Normal	88 (34.1)	
*IGHV mutation status*		
Positive	103 (39.9)	
Negative	155 (60.1)	
*ZAP70 *(*N* = 110)		
Positive	22 (20.0)	
Negative	88 (80.0)	
*CD38 *(*N* = 188)		
Positive	45 (23.4)	
Negative	143 (76.1)	

Q1: quartile 1; Q3: quartile 3; CLL-IPI: chronic lymphocytic leukemia international prognostic index.

**Table 3:** Overall survival and time to first therapy data of the MDACC PI, the CLL-IPI, and the Barcelona-Brno biomarkers only prognostic model risk groups.

MDACC PI						
Group	*N*	Median OS (95% CI)	5-year OS	10-year OS	HR (95% CI)	*p*

Low risk	160	238.5 (146–330.0)	98.4	91.6	1 (ref)	
Intermediate risk	302	142 (132–151.9)	79.6	61.6	4.8 (2.6–9.1)	<0.0001
High risk	21	63.5 (52.6–74.4)	51.1	15.3	5.3 (0–9.2)	<0.0001

Group	*N*	Median TTFT (95% CI)	5-year TTFT	10-year TTFT	HR (95% CI)	*p*

Low risk	160	133.0 (110.8–155.3)	20.3	47.5	1 (ref)	
Intermediate risk	302	81 (46.6–115.4)	44.9	55.7	2.2 (1.5–3.1)	<0.0001
High risk	21	2.3 (0.3–4.4)	93.7	93.7	4.4 (3.2–6)	<0.0001

CLL-IPI						

Group	*N*	Median OS (95% CI)	5-year OS	10-year OS	HR (95% CI)	*p*

Low risk	126	238.5 (147–330)	93.6	89.6	1	
Intermediate risk	79	144.8 (127.8–161.8)	87.6	74.5%	3.2 (1.5–6.7)	<0.0001
High risk	49	73.7 (56.6–90.7)	67.8	26.8	2.2 (2.1–4.8)	<0.0001
Very high risk	7	31.8 (21.2–42.4)	28.6	NE^*∗*^	4.4 (3.2–6)	<0.0001

Group	*N*	Median TTFT (95% CI)	5-year TTFT	10-year TTFT	HR (95% CI)	*p*

Low risk	126	Not reached	15.8	29.4	1 (ref)	
Intermediate risk	79	52.9 (34.5–71.3)	55	79.1	4.4 (2.7–7.2)	<0.0001
High risk	46	7.6 (5–10)	93.4	93.4	3.7 (2.8–4.9)	<0.0001
Very high risk	7	9.6 (0–21)	NE^*∗∗*^	NE^*∗∗*^	2.2 (1.6–3.2)	<0.0001

Barcelona-Brno PI						

Group	*N*	Median OS (95% CI)	5-year OS	10-year OS	HR (95% CI)	*p*

Low risk	145	238.5 (146.8–330)	90.7	86	1 (ref)	
Intermediate risk	91	131.9 (95–167.9)	81.4	61.1	4.34 (2.85–6.63)	<0.0001
High risk	22	Not reached	66.2	53	9.37 (5.12–17.14)	<0.0001

Group	*N*	Median TTFT (95% CI)	5-year TTFT	10-year TTFT	HR (95% CI)	*p*

Low risk	142	182.2 (83.9–280.4)	19.9	35.8	1 (ref)	
Intermediate risk	91	31.5 (14.4–48.5)	64.7	82.4	2.95 (1.57–5.52)	<0.0001
High risk	21	9.8 (2.4–17.2)	91.6	91.6	4.24 (1.67–10.97)	<0.0001

NE: not evaluable. NE^**∗**^: last control in month 94. NE^**∗∗**^: last control in month 45. MDACC: MD Anderson Cancer Center. CLL-IPI: chronic lymphocytic leukemia international prognostic index.

## Supplementary Materials

Supplementary Materials Table S1: univariate and multivariate Cox regression analyses of the variables included in the MDACC score. Table S2: univariate and multivariate Cox regression analyses of the variables included in the CLL-IPI score. Figure S1: ROC curve for MDACC PI. (A) Overall survival. (B) Time to first therapy. Figure S2: ROC curve for CLL-IPI. (A) Overall survival. (B) Time to first therapy. Figure S3: ROC curve for Barcelona-Brno biomarkers only prognostic model. (A) Overall survival. (B) Time to first therapy.
