# Supplementary material for: The International Prognostic Index for Patients with Chronic Lymphocytic Leukemia Has the Higher Value in Predicting Overall Outcome Compared with the Barcelona-Brno Biomarkers Only Prognostic Model and the MD Anderson Cancer Center Prognostic Index
Source: Biomed Res Int. 2018 Mar 15;2018:9506979. doi: 10.1155/2018/9506979 (PMC5874971; doi:10.1155/2018/9506979)
Supplement: Supplementary Materials — Table S1: univariate and multivariate Cox regression analyses of the variables included in the MDACC score. Table S2: univariate and multivariate Cox regression analyses of the variables included in the CLL-IPI score. Figure S1: ROC curve for MDACC PI. (A) Overall survival. (B) Time to first therapy. Figure S2: ROC curve for CLL-IPI. (A) Overall survival. (B) Time to first therapy. Figure S3: ROC curve for Barcelona-Brno biomarkers only prognostic model. (A) Overall survival. (B) Time to first therapy. [file 9506979.f1.pdf]

SUPPLEMENTARY INFORMATION

Univariate and multivariate analyses

We analyzed the association of the prognostic factors of each index with OS, as well as the HR of the univariate and multivariate analyses.

Regarding the univariate analysis of the sample used for the validation of the MDACC index, significance was shown for age, β2-microglobulin, absolute lymphocyte count, number of nodal regions affected and Rai stage. In the sex variable, a trend close to statistical significance was observed (p = 0.0067). However, in the multivariate analysis, significance was shown for sex but not for absolute lymphocyte count (p = 0.111). The factor that showed the greatest impact in the multivariate analysis was the pooling of patients in 0/I/II Rai stage *vs* III/IV Rai stage (HR = 3.1). A more comprehensive explanation of these analysis can be observed in Table S1.

Table S1. Univariate and multivariate Cox regression analysis of the variables included in the MDACC score.

	Median OS (months) (CI 95%)	P	Univariate Cox regression		Multivariate Cox regression	
			HR (CI 95%)	P	HR (CI 95%)	P
**Age (years)**						
<50	238.5 (0-477.1)	0.007	1.055 (1.032-1.078)	<0.0001	1.061 (1.035-1.086)	<0.0001
50-65	173.2 (NE)		*for 1 year increase*			
>65	142 (111.1-172.8)					
**Sex**						
Male	147.3 (111-183.6)	0.067	1.521 (0.971-2.385)	0.069	1.636 (1.016-2.636)	0.043
Female	194.5 (126.5-262.5)		1 (ref)			
**β2-microglobulin (mg/L)**						
Normal	238.5 (NE)	<0.0001	1.276 (1.209-1.346)	<0.0001	1.137 (1.055-1.225)	0.001
1-2 x ULN	143.5 (102.2-184.8)		*for 1mg/dL increase*			
>2 x ULN	63.5 (NE)					
**Lymphocyte count ($x10^9$/L)**						
<20	194.5 (137.3-251.8)	<0.0001	1.004 (1.002-1.006)	<0.0001	1.002 (1.000-1.005)	0.111
20-50	143.5 (63-224.1)		*for $1x10^9$ increase*			
>50	76.2 (44.9-107.5)					
**Enlarged node regions**						
≤2	194.5 (148.9-240.2)	<0.0001	1 (ref)	<0.0001	2.454 (1.396-4.313)	0.002
>2	73.9 (62.6-85.2)		3.700 (2.333-5.868)			
**Rai staging system**						
0-2	194.5 (145-244)	<0.0001	1 (ref)	<0.0001	3.108 (1.471-6.566)	0.003
3-4	71.5 (23.2-119.7)		5.770 (3.205-10.389)			

OS, overall survival; CI95%, 95% confidence interval; HR, hazard ratio; NE, not evaluable; ULN, upper limit normal; MDACC: MD Anderson Cancer Center.

In the univariate analysis of the CLL-IPI sample, β2-microglobulin, 17p-, *IGHV* mutational state, Rai stage and Binet stage were statistically significant. Only the comparison between patients ≤65 vs>65 years old did not obtain statistical significance ($p = 0.171$). However, in the multivariate analysis, no association between β2-microglobulin and OS ($p = 0.268$) was observed, but there was association when age was quantitatively analyzed. The factor with the greatest impact was 17p- (HR = 9.396). Details of these analyses can be seen in Table S2.

Table S2. Univariate and multivariate Cox regression analysis of the variables included in the CLL-IPI score.

	Median OS (months) (CI 95%)	P	Univariate Cox regression		Multivariate Cox regression	
			HR (CI 95%)	P	HR (CI 95%)	P
**Age (years)**						
<65	238.5 (126.4-350.6)	0.171	1.031 (1.003-1.059)	0.028	1.047 (1.016-1.078)	0.003
≥65	Not reached		*for 1 year increase*			
**β2-microglobulin (mg/L)**						
≤ 3.5	238.5 (129.9-347.2)	<0.0001	1.510 (1.273-1.791)	<0.0001 (n=237)	1.133 (0.909-1.412)	0.268
>3.5	71.5 (58.1-84.8´9		*for 1mg/dL increase*			
**Del 17p-**						
Yes	31.8 (23.5-40.0)	<0.0001	11.478 (4.087-32.236)	<0.0001	9.396 (3.159-27.943)	<0.0001
Not	238.5 (129.4-347.6)		1 (ref)			
***IGHV* mutation status**						
Mutated	238.5 (146.8-330.2)	<0.0001	1 (ref)	<0.0001	2.066 (1.003-4.136)	0.040
Non-mutated	131.9 (92.4-171.4)		3.141 (1.740-5.673)			
**Rai staging system**						
0	Not reached	<0.0001	1 (ref)	<0.0001	5.256 (2.541-11.288)	<0.0001
1-2	105.8 (31.8-179.8)		5.909 (3.080-11.337)			
3-4	71.5 (NE)		9.038 (2.995-27.274)			

OS, overall survival; CI95%, 95% confidence interval; HR, hazard ratio; NE, not evaluable; ULN, upper limit normal; CLL-IPI: Chronic Lymphocytic Leukemia International Prognostic Index.

Figure S1. ROC curve for MDACC PI. (A) Overall Survival. (B) Time to First Therapy.

(A)

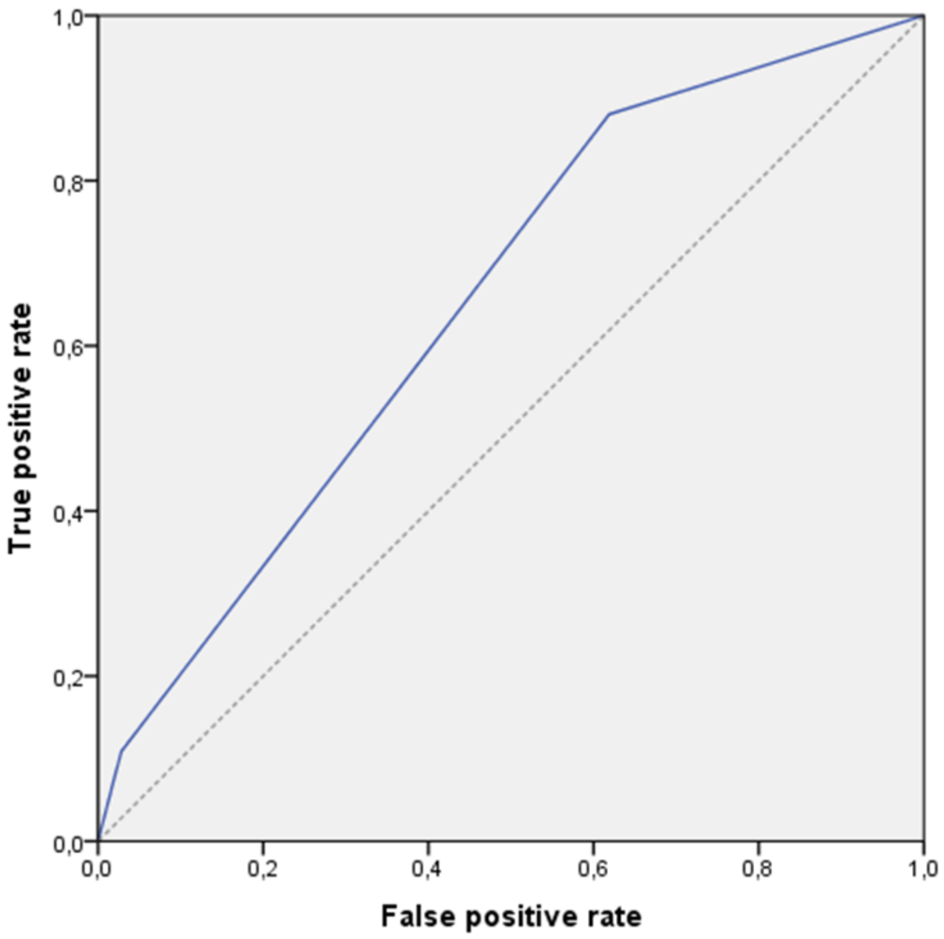

(B)

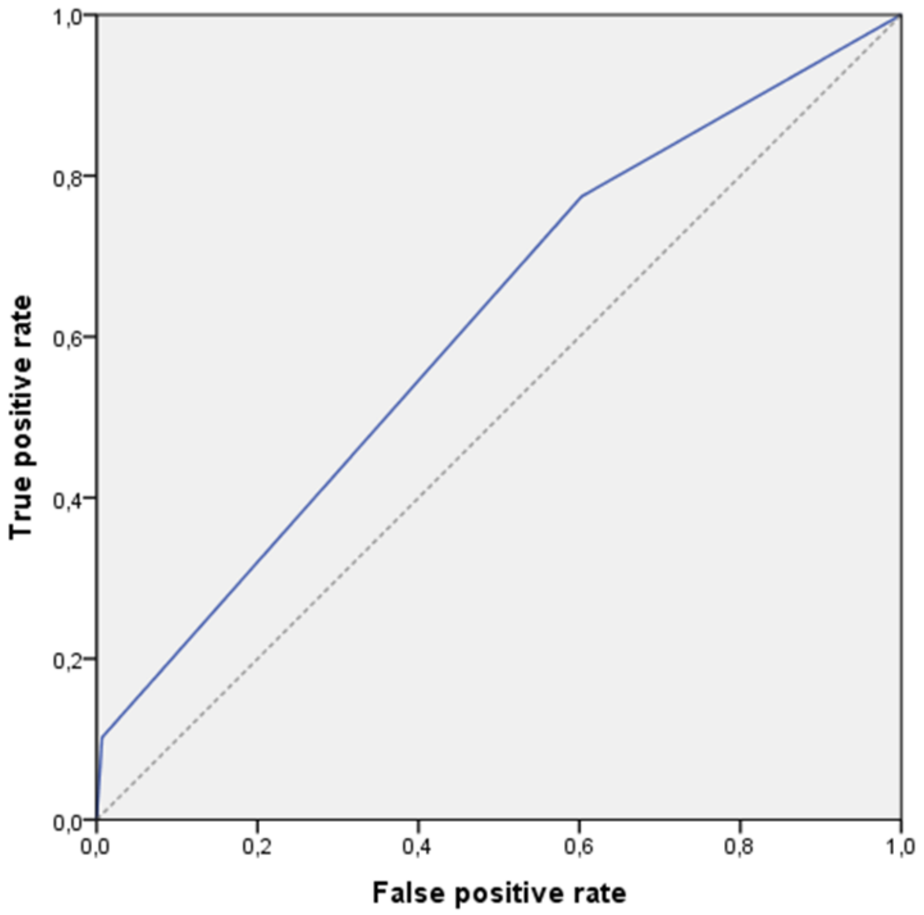
True positive rate
False positive rate
1,0
0,8
0,6
0,4
0,2
0,0
0,0
0,2
0,4
0,6
0,8
1,0

Figure S2. ROC curve for CLL-IPI. (A) Overall Survival. (B) Time to First Therapy.

(A)

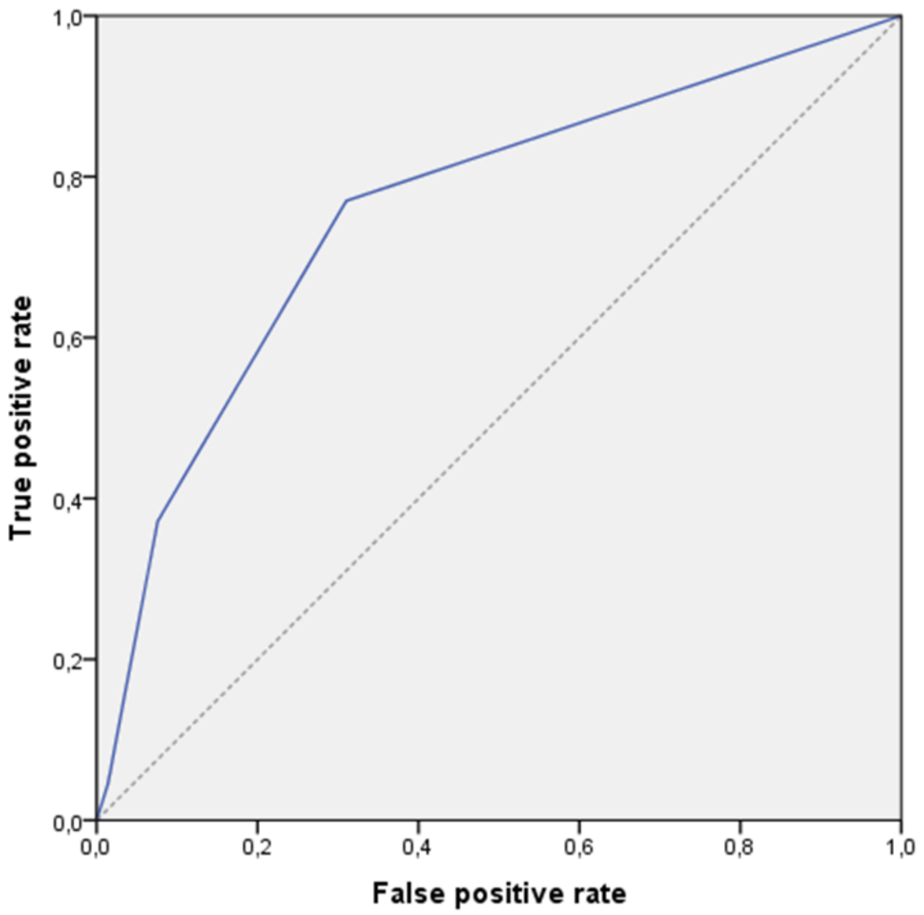

(B)

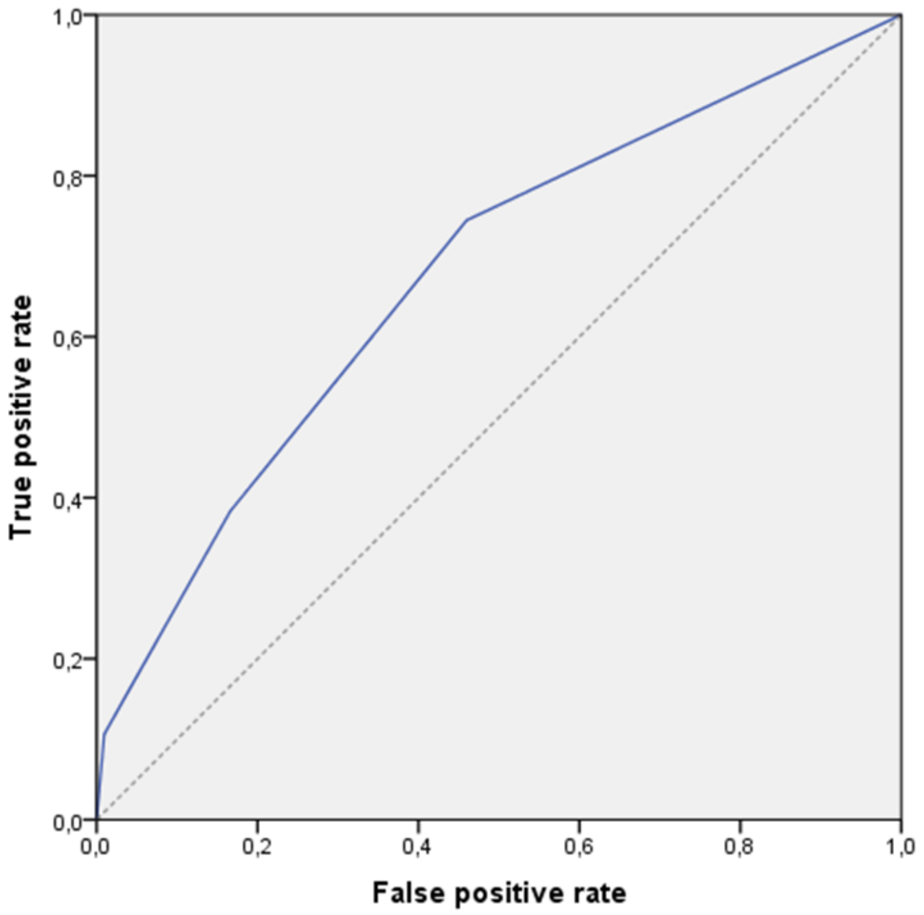
1,0
0,8
0,6
0,4
0,2
0,0
True positive rate
0,0
0,2
0,4
0,6
0,8
1,0
False positive rate

Figure S3. ROC curve for Barcelona-Brno biomarkers only prognostic model. (A) Overall Survival. (B) Time to First Therapy.

(A)

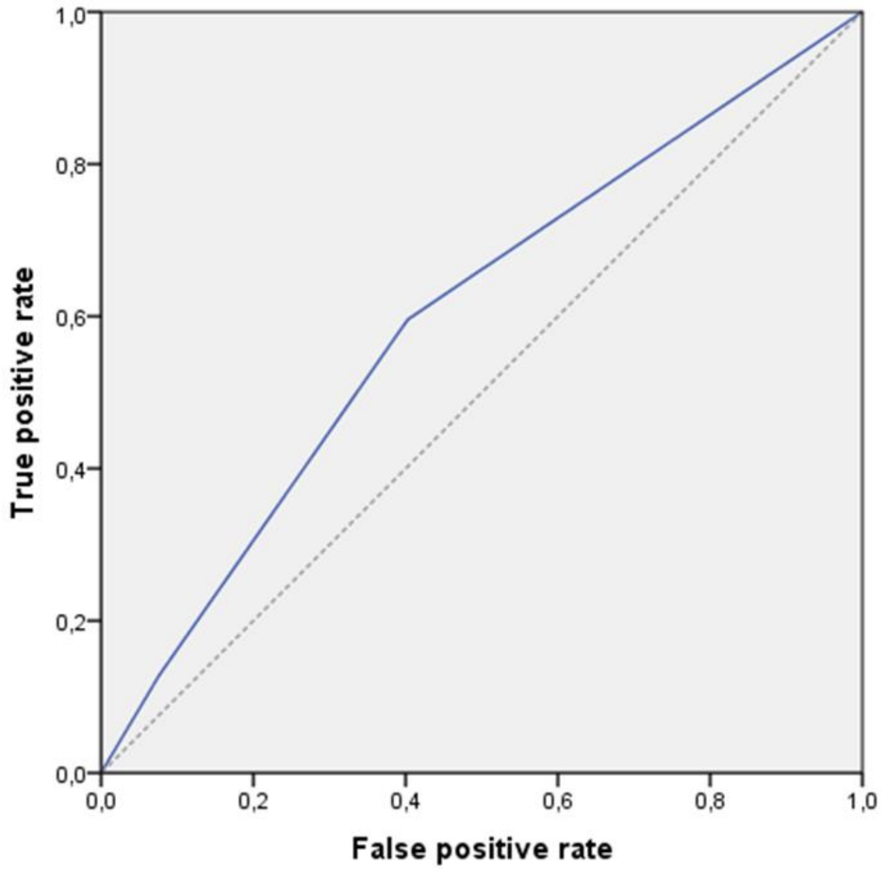

(B)

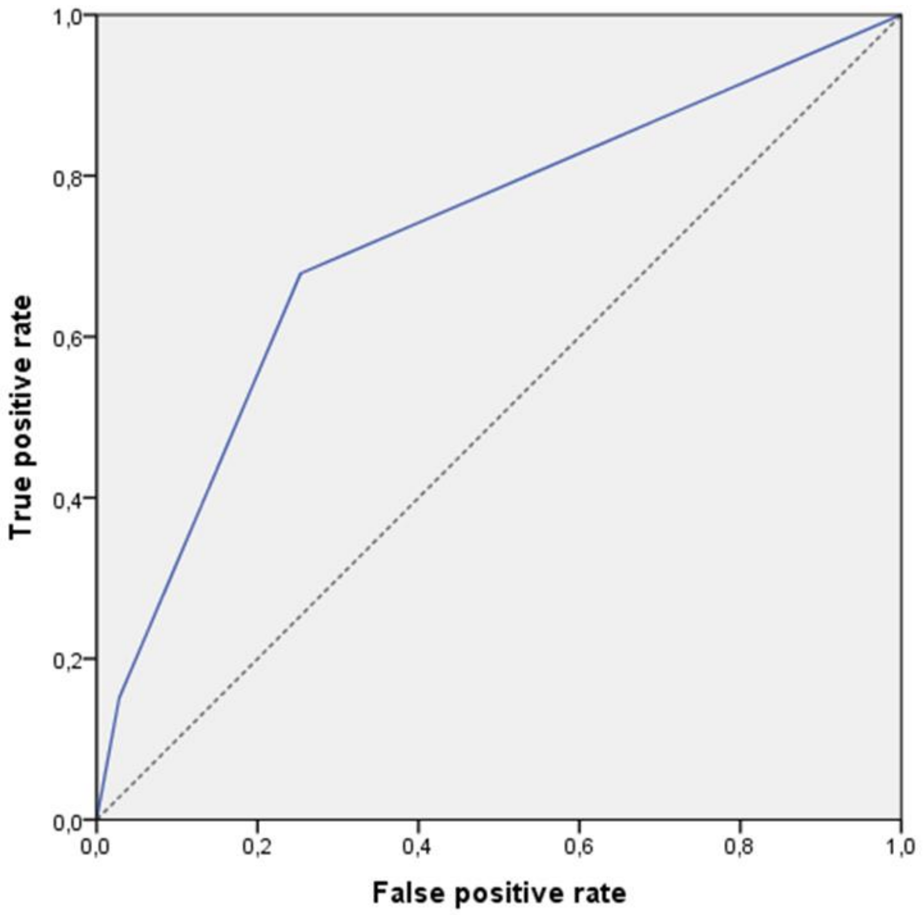

1,0
0,8
0,6
0,4
0,2
0,0
True positive rate
0,0
0,2
0,4
0,6
0,8
1,0
False positive rate
